# The gene patent controversy on Twitter: a case study of Twitter users’ responses to the CHEO lawsuit against Long QT gene patents

**DOI:** 10.1186/s12910-015-0049-1

**Published:** 2015-08-25

**Authors:** Li Du, Kalina Kamenova, Timothy Caulfield

**Affiliations:** Health Law Institute, Faculty of Law, University of Alberta, Room 464, Edmonton, AB T6G 2H5 Canada; Bachelor of Arts & Science Program (BAS), Trent University, Peterborough, ON Canada; Faculty of Law and School of Public Health, University of Alberta, Edmonton, AB Canada

## Abstract

**Background:**

The recent Canadian lawsuit on patent infringement, filed by the Children’s Hospital of Eastern Ontario (CHEO), has engendered a significant public debate on whether patenting genes should be legal in Canada. In part, this public debate has involved the use of social networking sites, such as Twitter. This case provides an opportunity to examine how Twitter was used in the context of this gene patent controversy.

**Methods:**

We collected 310 English-language tweets that contained the keyword “gene patents” by using TOPSY.com and Twitter’s built-in search engine. A content analysis of the messages was conducted to establish the users’ perspectives on both CHEO’s court challenge and the broader controversy over the patenting of human DNA. More specifically, we analyzed the users’ demographics, geographic locations, and attitudes toward the CHEO position on gene patents and the patentability of human genes in principle.

**Results:**

Our analysis has shown that messages tweeted by news media and health care organizations were re-tweeted most frequently in Twitter discussions regarding both the CHEO patent infringement lawsuit and gene patents in general. 34.8 % of tweets were supportive of CHEO, with 52.8 % of the supportive tweets suggesting that gene patents contravene patients’ rights to health care access. 17.6 % of the supportive tweets cited ethical and social concerns against gene patents. Nearly 40 % of tweets clearly expressed that human genes should not be patentable, and there were *no* tweets that presented perspectives favourable toward the patenting of human genes.

**Conclusion:**

Access to healthcare and the use of genetic testing were the most important concerns raised by Twitter users in the context of the CHEO case. Our analysis of tweets reveals an expectation that the CHEO lawsuit will provide an opportunity to clear the confusion on gene patents by establishing a legal precedent on the patentability of human genes in Canada. In general, there were no tweets arguing in favour of gene patents. Given the emerging role of social media in framing the public dialogue on these issues, this sentiment could potentially have an impact on the nature and tone of the Canadian policy debate.

## Background

With over 300,000,000 active users, Twitter has emerged as an important source of health-related information for both the general public and the research community [1, 2]. There is a growing body of scholarship that suggests that Twitter can have a significant impact on public perceptions as well as the framing of public policy issues and debates [3–7]. In this regard, opinions shared on Twitter can both reflect and shape public understandings and public discourse [8, 9]. Furthermore, Twitter is an important resource for research on public attitudes towards biomedicine and has the potential to facilitate knowledge exchange and public engagement with issues relating to health and illness [10]. To date, there has been no analysis of Twitter in the context of gene patents, one of the most contentious and longstanding policy issues in this area of research.

Canada is a jurisdiction where there has yet to be a high level of judicial scrutiny into the validity of gene patents [11]. Naturally occurring human genes are no longer patent-eligible in the United States after the Supreme Court’s decision in *Myriad* [12], but they are currently allowed under the existing Canadian patent law [13]. On November 3, 2014, a highly publicized lawsuit was launched in Canada by CHEO to invalidate patents for five genes associated with Long QT Syndrome (LQTS), a rare disorder of the heart’s electrical activity that may cause sudden, uncontrollable and dangerous arrhythmias [14, 15]. The LQTS gene patent holders requested that CHEO cease to conduct genetic testing for LQTS, and that patients’ blood samples be sent for analysis to licensed labs in the US. Currently, the cost of genetic testing in the US licensed labs is more than $4000 US dollars, with costs being half the price in Canadian hospitals [16, 17]. Given their potential impact on access to health care service within the Canadian system, news media, patient groups, and academics have long debated whether gene patents should be allowed in Canada [18, 19].

The CHEO case is the first legal action in Canada to challenge the patentability of human genes. Given its important policy implications, as well as the controversial nature of gene patenting more generally [20], the lawsuit has provided a good opportunity to examine how Twitter users represent their attitudes toward the CHEO lawsuit and gene patenting issues generally. In this paper, we analyze the content of tweets on both the CHEO case and gene patents more broadly, which were posted in the month immediately following news of CHEO having filed the lawsuit. We explore the Twitter users’ perspectives on the CHEO lawsuit and its societal implications, as well as their overall attitudes toward the issue of gene patents.

## Methods

We extracted the study sample using a combination of Twitter’s built-in search engine and TOPSY.com, a social search and analytics company that is partnered with Twitter, and which enables real-time searches for Twitter content. We collected all relevant tweets by using the keyword “gene patent”, and we excluded messages that did not mention the CHEO gene patent lawsuit or gene patents in general. The final dataset consisted of 310 English-language tweets that tweeted from November 3 — the date of the CHEO lawsuit was announced to December 3, 2014. We conducted a quantitative content analysis of the tweets to establish how CHEO’s court challenge and the broader controversy over the patenting of human DNA were represented. Tweets were coded for: 1) date of tweet; 2) sender information; 3) mentions of CHEO; 3) positive or negative attitude towards the CHEO lawsuit; and 4) the reasons for supporting or opposing the lawsuit.

Since content analysis is considered subjective, we asked an independent coder to code approximately 10 % of the tweets in our dataset (*n* = 34). An inter-coder reliability assessment was conducted using Cohen’ Kappa (*k*), which generated *k* scores on different coding categories in the range of 0.735-1.000, indicating substantial or almost perfect agreement based on the Landis & Koch’s benchmark for interpreting kappa [21].

## Results

**Fig. 1:**
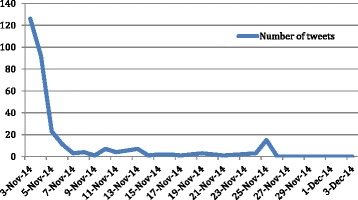
Number of tweets from 3 November, 2014 to 3 December, 2014

Twitter users reacted quickly to the news that the CHEO had launched a legal action over gene patents. The peak (*n* = 126) occurred on 3 November 2014, the first day that the hospital announced the gene patent lawsuit. The number of tweets decreased (*n* = 92) on 4 November 2014, and then dropped to less than seven tweets daily from November 7 to 24 (See Fig. 1). In terms of the geographic origin of the tweets, most senders (*n* = 166) were from Canada, with a comparatively small number of tweets (*n* = 29) posted by Twitter users in the United States. Over 60 % of the tweets (*n* = 191) were sent out by individuals. Members of the general public (i.e., Twitter users that did not specify their occupations or affiliations) were the majority, making up 32.9 % of the message senders (*n* = 102), whereas academics accounted for 15.8 % (*n* = 49) of the senders. For tweets that were sent by organizations, the following distribution was observed: advocacy and non-profit groups, 33.6 % (*n* = 40); news media, 31.9 % (*n* = 38); and health institutions, 17.6 % (*n* = 21) (Table 1).

**Table 1:** Demographics of the users

Senders	Number of sources	Percentage
Individuals	191	61.6(%)
Members of the public	102	53.4
Academics	49	25.7
Journalists	14	7.3
Clinical practitioners	12	6.3
IP lawyers	12	6.3
Industry representatives	2	1.0
Organizations	119	38.4(%)
Advocacy/non-profit groups	40	33.6
News media	38	32.0
Health institutions	21	17.6
Law firms	14	11.8
Academic institutions	6	5.0

We found that the most frequently re-tweeted messages (see Table 2) were headlines of news reports or articles published on news media websites and the majority of these tweets had incorporated web links to the news reports or articles. For example, the most popular re-tweet (*n* = 36), “How a gene-patent test case will help both patients and inventors” was the title of a review article published in *The Globe and Mail*. This article was authored by three Canadian university professors, including Richard Gold, a law professor at McGill University, who was providing pro bono legal services to Gilbert’s LLP, the law firm representing the CHEO in the Long QT gene patent lawsuit [22]. Similarly, other commonly re-tweeted messages, such as: “Ontario hospital launches lawsuit against owners of gene patent;” “No one should be able to patent human DNA;” and “Gene patent lawsuit aims to clear up confusion in Canada” are all news headlines that appeared on major news media websites.

**Table 2:** Content of frequently re-tweeted messages

Content of the tweet	Re-tweeted times
“How a gene-patent test case will help both patients and inventors”	36
“Ontario hospital launches lawsuit against owners of gene patent”	28
“No one should be able to patent human DNA”	19
“Gene patent lawsuit aims to clear up confusion in Canada”	15
“CHEO launches legal challenge of gene patent in order to protect patient care”	11

**Table 3:** Reasons for supporting CHEO

Arguments supporting the CHEO hospital	Number of tweets
• Protect patient care (e.g., access to diagnostics)	57
• It’s morally wrong to own/patent genes	19
• Lawsuit can help clear confusion on gene patents	15
• Saving $200,000 in health care cost annually	1
• US gene test monopoly	1
Arguments against gene patents in principle	
• Negative impact on patient care	25
• It’s morally wrong to own gene patents, or patient sharing of life-saving information	23
• Discovery of genes is not invention	13
• Gene patents invalidated in the US	3
• Patenting human genes is the same as owing humans	2
• Genetic test monopoly	1

There were 83.5 % tweets (*n* = 259) that mentioned the CHEO’s gene patent lawsuit. Our data showed that 34.8 % of tweets (*n* = 108) were supportive of CHEO, whereas the overall tone of 48.7 % of tweets (*n* = 151) was neutral or descriptive. When all tweets were taken into consideration, including those that did not mention the CHEO case, 38.7 % (*n* = 120) opposed the patenting of genes and the remaining tweets (*n* = 190) were neutral in tone. It is worth noting that no tweet explicitly supported gene patents. Twitter users’ most commonly stated reason for supporting CHEO was the need to protect patients’ access to diagnostics and health care, as indicated by the variable – 18.4 % (*n* = 57). Other two frequently stated reasons for supporting the hospital’s decision to file the lawsuit included: 1) claims that it’s morally wrong to own or patent human genes (6.1 % (*n* = 19)); and 2) claims that human DNA is not patentable (4.8 % (*n* = 15)) (see Table 3).

## Discussion

Our research indicates that most frequently re-tweeted messages were tweets posted by news media organizations (e.g., CBC Health New, CTV News, *The Global and Mail*) and health care institutions (e.g., CHEO). While tweeting news on CHEO’s lawsuit was the major characteristic of user activity, arguments against the patenting of human genes were a common theme (e.g., claims that gene patents will hamper patients’ access to diagnostics). Links to news media sites that discussed gene patent controversies were widely shared and included in many tweets. Given the existing literature on framing and the impact of social media, these findings hint at the possible impact of Twitter on the public’ perceptions of the gene patents controversy [23].

Although 61 % of tweets did not explicitly express the user’s personal attitudes toward gene patents, our analysis has shown that nearly 40 % of users did argue against gene patenting and that there were no tweets that explicitly opposed CHEO’s decision to challenge LQTS patents in court. In fact, 34.8 % tweets were supportive of the hospital. Most significant, there were *no* tweets arguing in favour of gene patents in general. These findings are consistent with previous studies that have shown negative public attitudes towards biotechnology patents [23–25]. Although Twitter posts are limited to 140 characters, there were some nuanced perspectives shared on Twitter when debating whether human genes should be patented. For example, the frequently re-tweeted message, “How a gene-patent case will help both patents and inventors”, incorporated a link to a review article that provided an analysis that argued that the existing Canadian gene patent system restricts access to diagnostics and data, which will negatively impact both patient health care and the development of genomics-based biomedical research [26]. Some tweets suggested that the CHEO case provides an opportunity to clarify the existing confusion surrounding gene patents in Canada.

With regard to rationales that were provided to support a position against patenting genes, several legalistic arguments, such as “it should not be legal to patent gene mutations”, and the “discovery of genes is not an invention”, were presented, but they were not significant themes. The US Supreme Court’s ruling in *Myriad* was also mentioned by some Twitter users [27]. The most common argument for opposing gene patents involved concerns associated with access to health care. Many Twitter users seemed worried about the possibility of private companies’ using patents to limit patients’ access to patient care, with more specific concerns focusing on how patents may affect the rare disease community, such as patients with LQTS. The findings also reveal some users’ ethical concerns about the patenting of human genetic materials (e.g., that patenting human gene is morally unacceptable and that naturally occurring genes should not be patentable). While we can only speculate about reasons that are driving Twitter users’ comments, studies have shown that media portrayals of gene patenting debates are relevant [24] and surveys have found a general negative attitude toward the idea of patenting human genes [28]. Furthermore, there is an ongoing public debate about how best to ensure access to expensive treatments for rare diseases [29].

## Conclusion

Although the CHEO news story did not become a viral global news story on Twitter, it did receive immediate attention by Twitter users, especially in Canada. Our content analysis of Twitter users’ responses to the breaking news indicates that individuals who have reservations about gene patents do share those sentiments on Twitter. While many tweets focused on spreading the message about the pending lawsuit, users have also articulated specific reasons for opposing the patenting of human genes and have speculated about the potential impact of the lawsuit on patients and IP rights holders in Canada. In general, Twitter users did not post tweets in support of gene patents. Given the emerging role of social media in the framing of public dialogue, this sentiment could have an impact on the nature and tone of the Canadian policy debate.

## Abbreviations

CHEO: Children’s Hospital of Eastern Ontario
LQTS: Long QT Syndrome
